# Associations of Exposure to Air Pollution with Insulin Resistance: A Systematic Review and Meta-Analysis

**DOI:** 10.3390/ijerph15112593

**Published:** 2018-11-20

**Authors:** Jiajia Dang, Mengtong Yang, Xinge Zhang, Haotian Ruan, Guiyu Qin, Jialin Fu, Ziqiong Shen, Anran Tan, Rui Li, Justin Moore

**Affiliations:** 1 School of Health Sciences, Wuhan University, 115 Donghu Road, Wuhan 430071, China; estelladd@outlook.com (J.D.); yangmt08@163.com (M.Y.); ylcz2920@126.com (X.Z.); novamadeus@icloud.com (H.R.); yuu_qin@163.com (G.Q.); Fjl708326@163.com (J.F.); zq_shen666@163.com (Z.S.); chloetar@hotmail.com (A.T.); 2 Department of Family & Community Medicine, Wake Forest School of Medicine, Medical Center Boulevard, Winston-Salem, NC 27157, USA; 3 Department of Epidemiology & Prevention, Wake Forest School of Medicine, Medical Center Boulevard, Winston-Salem, NC 27157, USA; 4 Department of Implementation Science, Wake Forest School of Medicine, Medical Center Boulevard, Winston-Salem, NC 27157, USA

**Keywords:** air pollution, insulin resistance, meta-analysis

## Abstract

In this article, we review the available evidence and explore the association between air pollution and insulin resistance (IR) using meta-analytic techniques. Cohort studies published before January 2018 were selected through English-language literature searches in nine databases. Six cohort studies were included in our sample, which assessed air pollutants including PM_2.5_ (particulate matter with an aerodynamic diameter less than or equal to 2.5 μm), NO_2_(nitrogen dioxide), and PM_10_ (particulate matter with an aerodynamic diameter less than 10 μm). Percentage change in insulin or insulin resistance associated with air pollutants with corresponding 95% confidence interval (CI) was used to evaluate the risk. A pooled effect (percentage change) was observed, with a 1 μg/m^3^ increase in NO_2_ associated with a significant 1.25% change (95% CI: 0.67, 1.84; I^2^ = 0.00%, *p* = 0.07) in the Homeostasis Model Assessment of Insulin Resistance (HOMA-IR) and a 0.60% change (95% CI: 0.17, 1.03; I^2^ = 30.94%, *p* = 0.27) in insulin. Similar to the analysis of NO_2_, a 1 μg/m^3^ increase in PM_10_ was associated with a significant 2.77% change (95% CI: 0.67, 4.87; I^2^ = 94.98%, *p* < 0.0001) in HOMA-IR and a 2.75% change in insulin (95% CI: 0.45, 5.04; I^2^ = 58.66%, *p* = 0.057). No significant associations were found between PM_2.5_ and insulin resistance biomarkers. We conclude that increased exposure to air pollution can lead to insulin resistance, further leading to diabetes and cardiometabolic diseases. Clinicians should consider the environmental exposure of patients when making screening and treatment decisions for them.

## 1. Introduction

Exposure to air pollution can influence human health in a variety of ways, leading to a dramatic risk in morbidity and mortality [1]. Epidemiological evidence has shown an increasing influence of air pollution on health, with air pollution becoming the largest environmental risk factor for a variety of chronic diseases [2]. The main air pollutants include particulate matter (PM), ozone, carbon monoxide, nitrogen dioxide, and sulfur dioxide [1]. Particulate matter pollutants are complex mixtures of solid and liquid particles of organic and inorganic substances suspended in the air, and a large fraction of PM is organic [3,4,5]. The World Health Organization (WHO) Air Quality guidelines (AQGs) have recommended the lowest concentration values of PM as PM_2.5_ (PM with an aerodynamic diameter less than or equal to 2.5 μm) of no more than 10 μg/m^3^ annual mean, 25 μg/m^3^ 24-h mean, and PM_10_ (PM with an aerodynamic diameter less than 10 μm) of no more than 20 μg/m^3^ annual mean, 50 μg/m^3^ 24-h mean. [1] According to a survey, more than three-fourths of the world’s population lives in areas with values over the annual air quality standard for PM_2.5_ set by the WHO (>10 μg/m^3^) [6,7]. With the intensification of air pollution, chronic diseases related to air pollution are attracting increasingly more attention. Numerous epidemiological investigations have shown that air pollutants are associated with some common chronic diseases, such as hypertension [8] and diabetes mellitus [9,10]. Other studies have also suggested that insulin resistance (IR) can be affected by air pollution [11,12,13].

IR is a condition characterized by decreased tissue sensitivity to the action of insulin [14,15,16]. It refers to various factors weakening the ability of insulin to uptake and utilize glucose. In parallel with this process, the body compensates through excessive secretion of insulin to maintain the stability of blood glucose. Homeostasis Model Assessment of Insulin Resistance (HOMA-IR: HOMA−IR=fasting insulin(μU/mL)×fasting glucose(mmol/l)/22.5) has been used to estimate the level of IR in the existing studies [17]. Elevated IR is defined as a HOMA-IR ≥2.6 according to standard cutoff points [18]. In addition, the changes in insulin, glucose, HbA1c, and leptin are also observed.

IR can increase the risk of developing type 2 diabetes and is considered an independent predictor of type 2 diabetes [19,20]. Genetics, excessive eating, and reduced physical activity are major risk factors or causes of the disease [21,22]. However, an increasing number of studies have reported that air pollution is also an important risk factor for diabetes [23,24]. Moreover, air pollution and IR as a precursor to type 2 diabetes has been shown to have a positive association [12,25]. IR also plays a significant role in obesity, cardiovascular disease, and metabolic syndrome [26,27,28,29]. Owing to the aging of the population and the increase in adverse environmental factors, the prevalence of these diseases is increasing rapidly. Growing evidence has suggested an association between air pollution and IR [30,31], but the results are equivocal. Some clinical studies have shown that high levels of ambient and traffic-related air pollution are associated with increased HOMA-IR in children and adults [31,32]. For example, Toledo-Corral et al. suggested that PM_2.5_ exposure was linked to higher fasting insulin, fasting glucose, acute insulin response to glucose, and lower insulin sensitivity [33]. Jin et al. found that air pollutants PM_10_ and nitrogen dioxide (NO_2_) was positively associated with insulin and HOMA index in elderly Koreans [12]. These findings suggest that air pollutants are positively associated with IR. However, in other long-term exposure studies, although air pollutants were positively associated with the level of glucose, this relationship was not found in either insulin or HOMA-IR [14,34]. For instance, Ward-Caviness et al. found that people who lived closer to the roadways had higher fasting blood glucose levels but no difference in HOMA-IR [34]. In addition, in studies by Brook et al. and Li et al., PM_2.5_ was associated with higher levels of HOMA-IR and glucose but did not have a relationship with insulin [11,35]. Thus, the magnitude of the association between air pollution and IR remains unclear.

The existing evidence from mechanistic studies have indicated possible biological pathways related to NO_2_, PM, and IR. Although the toxicity of air pollutants is different, they are all considered effective oxidants, acting directly on lipids and proteins or indirectly through the activation of intracellular oxidation pathways [36,37]. NO_2_ has been proven to trigger oxidative stress, inflammation, and biological pathways that promote IR [38]. The effect of NO_2_ on the level of oxidized low-density lipoprotein (LDL) in adolescents [39] was reported in a study. Some studies have reported that exposure to PM and NO_2_ leads to elevated biomarkers of inflammation [14,40,41]. Studies have also shown PM-induced oxidation potential of proteins and lipids in young adults [42]. This suggests that oxidative stress caused by air pollution may play a role in IR.

The adverse effects of air pollution were also observed on c-reactive protein, TNF-α, and interleukin-1β in children [13]. One explanation for the mechanism of IR is that oxidative stress-induced endoplasmic reticulum stress pathway could lead to activation of c-Jun N-terminal kinase and ultimately impair insulin signaling in muscle tissue [43].

Besides, inflammation and the alterations in glucose metabolism are also potential mechanisms for the relationship between PM and IR [44]. In animal experiments, PM-mediated elevation of blood glucose was observed in mice in the normal diet and high-fat diet groups [25,45]. The adverse reaction of insulin signal in liver tissue is the basis of IR pathogenesis. The reduced Akt phosphorylation in liver, white adipose tissue, and skeletal muscle in response to insulin stimulation indicates the development of insulin resistance in multiple organs [44]. PM-activated toll-like receptors can also induce metabolic pathways, including changes in glucose metabolism [46].

The purpose of the present study was to review the available evidence of epidemiological observational studies concerning the relationship between air pollution and IR, systematically explore the relationship between the two using meta-analytic techniques, and provide evidence for the formulation of effective preventive measures. We hypothesized that exposure to air pollution could increase insulin resistance levels.

## 2. Materials and Methods

This meta-analysis was performed according to the Preferred Reporting Items for Systematic Reviews and Meta-Analyses (PRISMA) statement [47].

### 2.1. Inclusion Criteria

We included studies that met the following criteria: (1) studies exploring the direct association between air pollution and insulin resistance; (2) retrospective or prospective cohort studies; (3) studies with sufficient data for extraction, such as percentage change and 95% confidence intervals (95% CI); (4) articles written in English.

### 2.2. Search Strategy

**Figure 1:**
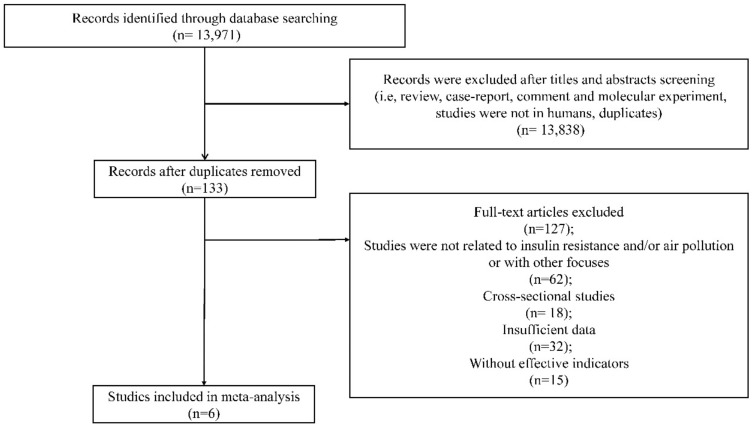
Flow chart of the study selection process.

We systematically searched nine English databases (PubMed, Embase, Web of Science, Nature, Science Direct, OVID, Springer, The Cochrane Library, and JAMA) for pertinent literature published before January 2018. PubMed, Embase, Web of Science, Nature, OVID, Springer, and The Cochrane Library were accessed from Wuhan University Library. Science Direct and JAMA were available on the official website. The search strategies were carried out on the basis of combinations of keywords concerning air pollution (“air pollution” OR “air pollutants” OR “particulate matter” OR “PM_10_” OR “PM_2.5_” OR “nitrogen dioxide” OR “NO_x_” OR “ozone” OR “soot” OR “smog” OR “carbon monoxide” OR “sulphur dioxide”) and insulin resistance (“insulin” OR “insulin secretion” OR “insulin resistance” OR “hyperinsulinism” OR “hyperinsulinemia” OR “insulin sensitivity” OR “beta cell” OR “glucose” OR “glycaemic” OR “HbA1c” OR “hyperglycaemia” OR “hyperglycemia”). The language was restricted to English. We downloaded all references identified from the databases into a reference manager (NoteExpress 3.2, Aegean Software, Beijing, China). The bibliography of all included studies, and those of previous reviews on the subject were examined for further relevant studies. The duplicates were deleted from the initial records using the software, and the remaining articles were sorted for eligibility using the following two steps: First, we reviewed titles and abstracts of all publications for eligibility. Then, we further evaluated the full texts for the remaining references that were labeled as potentially eligible (Figure 1).

### 2.3. Selection of Studies

There was no limit to the study population. Each study selection was performed by two independent investigators (Jiajia Dang and Mengtong Yang) in order to reduce the potential for selection bias, and a mutual decision was made together regarding whether or not it met the basic inclusion criteria. The disagreements were solved by negotiating with a third investigator (Xinge Zhang), and consensus was reached after discussion.

### 2.4. Data Extraction

The following information was extracted from each included study: author, published year, age, study country, study period, exposure, exposure assessment, outcome assessment, adjusted variable, statistical model and estimates (odds ratio (OR), regression coefficient (b), risk ratio (RR), or hazard ratio (HR)), and their corresponding 95% confidence interval (CI) or standard error. Two researchers (Jiajia Dang and Mengtong Yang) independently extracted the data from each study, and a third author (Xinge Zhang) adjudicated conflicts. We contacted the original authors to acquire the original data for studies that did not have enough data.

### 2.5. Quality Assessment

Two investigators (Jiajia Dang and Mengtong Yang) independently assessed the quality of each study. We solved any disagreements by discussion. We used the Newcastle–Ottawa scale (NOS) for cohort studies to assess the quality of individual studies [48]. There are three dimensions in this scale: selection of the study groups, comparability of the study groups, and outcome ascertainment. There are altogether eight questions raised in this scale, with a minimum of zero and a maximum of nine stars. The quality of the study is then graded as poor (0–3 stars), intermediate (4–6 stars), or high (7–9 stars) [48].

### 2.6. Statistical Analysis

We adopted percentage change of insulin resistance indicators as the effect size because most of the included studies used percentage change as effect amount. Percentage increase and percentage decrease are measures of percentage change, which is the extent to which a variable gains or loses intensity, magnitude, extent, or value. We produced forest plots to show percentage change and the estimation of the summary percentage change. We recalculated the coefficient estimates to reflect a 1 μg/m^3^ increase in PM_10_, PM_2.5_ and NO_2_, assuming a linear relationship within the considered range. The between-study heterogeneity was assessed by I^2^ statistics (no heterogeneity: I^2^ = 25%; moderate heterogeneity: I^2^ = 25–50%; large heterogeneity: I^2^ = 50–75%; extreme heterogeneity: I^2^ = 75–100%) and Q-test. The random effects model was utilized if the heterogeneity was statistically significant (I^2^ > 50% and *p* < 0.05); otherwise, the fixed effects model was adopted [49]. We also examined the influence of excluding each study and/or some specific studies to test the stability of our results [50]. Meta-regression was performed to identify participant age that can possibly explain the between-study variation in the reported percentage change [51]. Finally, we used funnel plot and Egger’s regression test to examine publication bias [52]. All these statistical analyses were carried out using R software (R Foundation for Statistical Computing, Vienna, Austria). All *p* values were two-tailed, and less than 0.05 was considered statistically significant.

## 3. Results

### 3.1. Literature Retrieval and Study Characteristics

The literature screening process is shown in Figure 1. Table 1 summarizes the characteristics of the included studies, all of which were cohort studies conducted in Germany (*n* = 3), the United States of America (*n* = 2), and Belgium (*n* = 1). The final sample included 11,656 participants, aged between 0 and 56.2. For cohort studies, the duration of follow-up ranged from 2 to 13 years.

The association between IR and PM_2.5_ (*n* = 6), NO_2_ (*n* = 5), PM_10_ (*n* = 4) was investigated in the included studies. One study explored the association of IR and the diameter of particulate matter between 2.5 and 10 μm (PM coarse) [14]. One study reported the association of per 500 m decrease in distance to major road with IR [13], and another one reported the association between traffic-related pollution and IR [53].

Among the six studies, two studies reported an association between NO_2_ [31] and traffic-related air pollution [14] and IR, respectively; one studied the association of air pollution [13] (including PM_2.5_, NO_2_, and PM_10_) exposure and risk of IR; and the last three studies examined the effect of elevated NO_2_ and PM_2.5_ on insulin homeostasis [40,41,53].

### 3.2. The Association of Exposure to PM_2.5_ and IR

**Figure 2:**
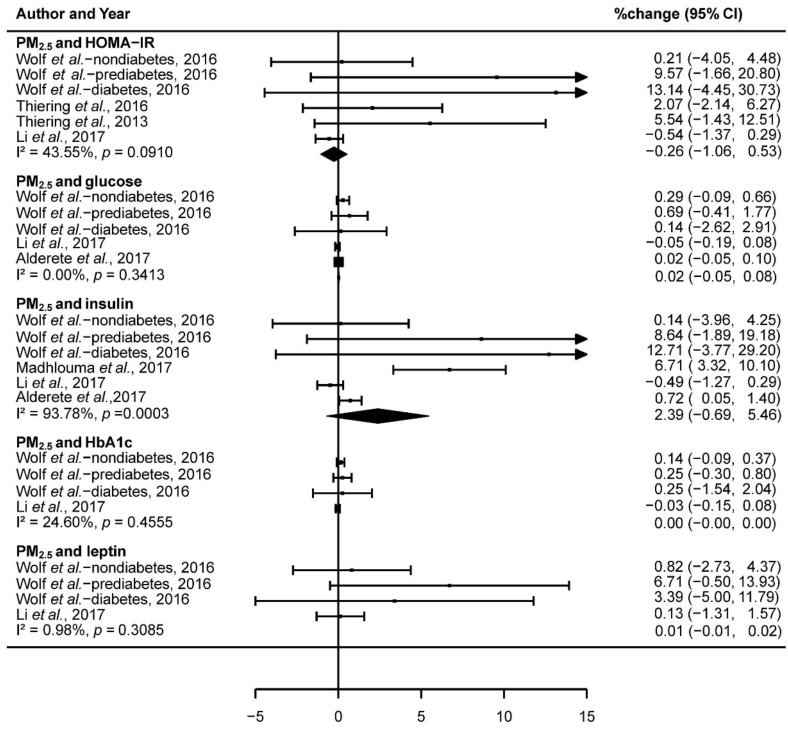
Forest plot showing the association between PM_2.5_ and insulin resistance.

We pooled data from included studies (dividing the study of Wolf et al. into three groups: nondiabetes, prediabetes, and diabetes) to assess the association of PM_2.5_, NO_2_, and PM_10_ with IR. Results showed that there was no significant association between PM_2.5_ and HOMA-IR (−0.26% change; 95% CI: −1.06,0.53; I^2^ = 43.55%, *p* = 0.09), glucose (0.02% change; 95% CI: −0.05,0.08; I^2^ = 0.00%, *p* = 0.34), insulin (2.39% change; 95% CI: −0.69,5.46; I^2^ = 93.78%, *p* < 0.001), HbA1c (0.00% change; 95% CI: −0.00,0.00; I^2^ = 24.60%, *p* = 0.46), and leptin (0.01% change; 95% CI: −0.01,0.02; I^2^ = 0.98%, *p* = 0.31; Figure 2). Heterogeneity analysis showed that, except for the exposure to PM_2.5_ with insulin, the other biomarkers (HOMA-IR, glucose, HbA1c, and leptin) did not have significant heterogeneity in the included studies (Figure 2).

### 3.3. The Association of Exposure to NO_2_ and IR

**Figure 3:**
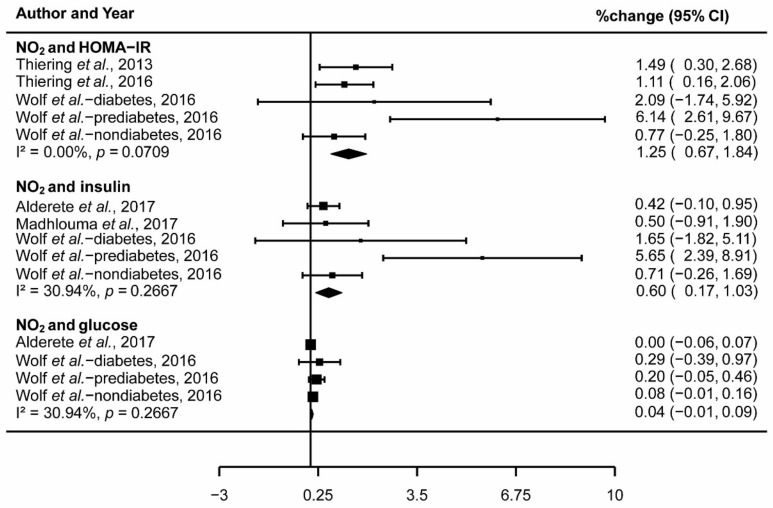
Forest plot showing the association between NO_2_ and insulin resistance.

The pooled effect percentage change) across studies reported the association between 1 μg/m^3^ increase in NO_2_ and 1.25% change in HOMA-IR (95% CI: 0.67,1.84; I^2^ = 0.00%, *p* = 0.07; Figure 3) and 0.60% change in insulin (95% CI: 0.17,1.03; I^2^ = 30.94%, *p* = 0.27; Figure 3). However, there was no significant association between NO_2_ and glucose (0.04% change; 95% CI: −0.01,0.09; I^2^ = 30.94%, *p* = 0.27; Figure 3). The meta-analysis of exposure to NO_2_ and IR did not have significant heterogeneity (Figure 3).

### 3.4. The Association of Exposure to PM_10_ and IR

**Figure 4:**
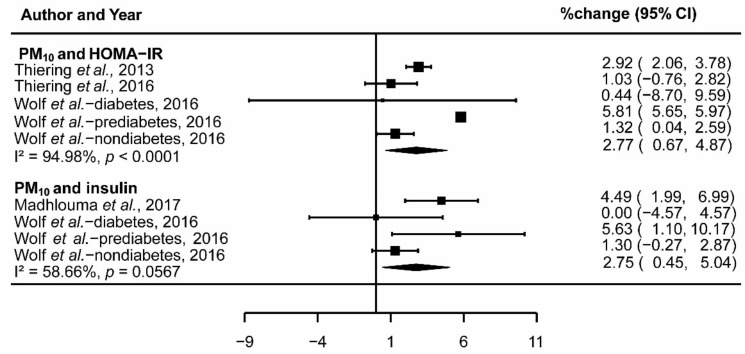
Forest plot showing the association between PM_10_ and insulin resistance.

Similar to the analysis of NO_2_, the results reported the association between 1 μg/m^3^ increase in PM_10_ and 2.77% change in HOMA-IR (95% CI: 0.67,4.87; I^2^ = 94.98%, *p* < 0.001; Figure 4) and 2.75% change in insulin (95% CI: 0.45,5.04; I^2^ = 58.66%, *p* = 0.057; Figure 4) after pooling three included studies. In addition, there was no significant association between PM_10_ and glucose, HbA1c, and leptin. While significant heterogeneity was found out in the group of exposure to PM_10_ and HOMA-IR, studies included in the group of PM_10_ and insulin did not demonstrate significant heterogeneity (Figure 4).

### 3.5. Sensitivity Analysis

We checked the influence of the removed data to the pooled estimates after deleting one single study from the pooled analysis each time. In addition to the studies of Li et al. [53] and Alderete et al. [40], the pooled estimates were generally robust for the exclusion of each study or some specific studies (Data not shown).

### 3.6. Meta-Regression

Meta-regression analyses showed that age was not a significant predictor of pooled estimates (percentage change in IR biomarkers) exposure to air pollution (Supplementary Table S1).

### 3.7. Publication Bias Analysis

**Figure 5:**
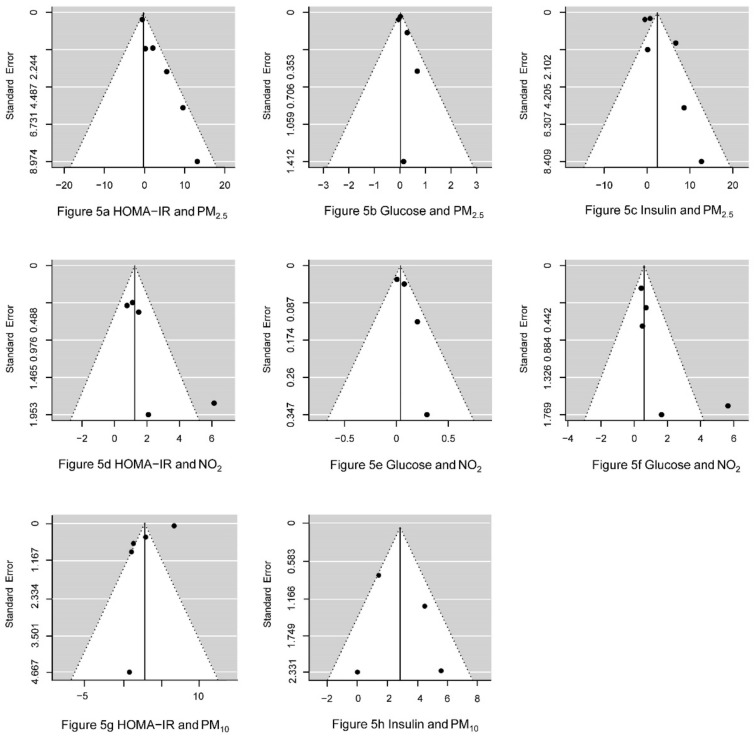
Funnel plot to explore publication bias for each pollutant.

The shape of the funnel plot (Figure 5) shows the asymmetry of the data points, suggesting that the loss of negative results produced by less precise studies might lead to a potential publication bias of PM_2.5_, NO_2_, and PM_10_. The plots show the observed outcomes (percentage change) on the horizontal axis against their corresponding standard errors for PM_2.5_, NO_2_, and PM_10_. Egger’s test revealed the presence of publication bias in the analysis of the relationship between PM_2.5_ and HOMA-IR (*p* = 0.004), PM_2.5_ and insulin (*p* = 0.03), NO_2_ and HOMA-IR (*p* = 0.02), and NO_2_ and insulin (*p* = 0.02) (Table 2).

## 4. Discussion

To the best of our knowledge, this is the first meta-analysis to systematically examine the relationship between air pollution and IR. The results showed that the exposure to NO_2_ and PM_10_ were associated with IR, demonstrating that the levels of NO_2_ and PM_10_ increased the risk of IR. However, there was no significant association between PM_2.5_ and the risk of IR.

The advantages of our research include comprehensive searches across multiple databases, a robust meta-analysis after adjusting the exposures (1 μg/m^3^ of PM_2.5,_ NO_2_ and PM_10_), and the large sample size and resulting statistical power. In addition, the design of the included cohort studies was more suggestive of a causal effect of air pollution and IR. Moreover, the process of data extraction and data analysis was rigorous and reproducible.

However, our research also has some limitations. First, there were a limited number of included studies that incorporated differences in research exposure assessment strategies. Cross-sectional studies were not included because we sought to establish the directionality of the relationship. Second, we found substantial heterogeneity for PM_10_ analyses and serious risk of bias for included studies, which reduced the credibility of the cumulative evidence. When analyzing the effects of PM_2.5_ exposure on insulin, the included studies were heterogeneous in the respects that the levels of exposure in various studies varied and that the assessment methods for exposure levels were not uniform. IR is affected by multiple factors, but the adjustment of confounding factors in the included studies was inconsistent, which might have been the reason for the heterogeneity of this meta-analysis. Besides, the outcome measures were all laboratory indexes. Although the measurement was convenient and the accuracy was satisfactory, the systematic error of the measuring instrument would have been inevitable, which might have also been a source of heterogeneity. Third, in this study, as an important adjusted factor, the wide age ranges might be one of the sources of heterogeneity. In addition, people with different ages have different sensitivity to air pollution and susceptibility to IR, but the results showed that age was not the cause of heterogeneity. It has been proven that higher insulin levels in early childhood are associated with increased risk of type 2 diabetes during adult life exposure to air pollutants [54]. However, diabetes does not occur in all patients with insulin resistance [14]. In addition, the limited number of included studies may have led to nonsignificant result. Fourth, we did not find any study from developing countries (India, China, and South America). In fact, air pollution in China is a significant public health burden, and the mean annual averages of PM_2.5_ and PM_10_ in 2013 were 72.4 and 118.5 μg/m^3^, respectively [55], which were nearly fivefold higher than the WHO standard of 10 μg/m^3^ and 20 μg/m^3^ for PM_2.5_ and PM_10_, respectively. Fifth, a single-pollutant model that did not account for the potential interaction between pollutants was used in most of our included studies. It is likely that a model of all possible sources of air pollution will be more accurate than a single pollutant model. Thus, other unmeasured variables, such as interactions between air pollutants and air pollutants species, may also have played an important role in explaining the observed heterogeneity. Sixth, the exposure assessment strategy varied from study to study and the majority of estimates were based on exposure levels of outdoor or circumjacent area. Therefore, there was no information about the impact of indoor air pollution on susceptibility to IR. Seventh, based on substantial heterogeneity tested out in some groups, random effects model was frequently conducted in our meta-analysis to pool the studies. However, this model has limitations in underestimating the statistical error and yielding overconfident conclusions [56]. Finally, the sample size for some air pollutants (e.g., PM_2.5_, NO_2_, and PM_10_) was not large enough. The above limitation could be sources of bias in this meta-analysis.

The possible effect of NO_2_ on IR [39,57] has been found in prior studies. Thiering et al. found that IR increased by 17.0% and 18.7% as the result of increased exposure to NO_2_ and PM_10_, respectively, and that the level of IR was greater in children [13]. Another study of 560 elderly people also showed that air pollutants such as O_3_ and NO_2_ were significantly associated with IR biomarkers in South Koreans [12]. In addition, several reviews have provided strong evidence that long-term exposure to high level PM_10_ increases the risk of IR [10,58]. A study consisting of 374 adolescents in Isfahan, Iran, reported PM_10_ concentration was related to IR [39]. A strong interaction between PM_10_ and IR [59] was also observed in another study. These studies are similar to our findings of the positive relationship between NO_2_, PM_10_, and IR. Several experiments have found the potential mechanism of the relationship between NO_2_, PM and IR. However, more toxicological research on this mechanism is needed to directly investigate whether NO_2_ promotes IR [60].

As for the relationship between PM_2.5_ and IR, quite a few studies have provided inconsistent evidence. One study of 25 American adults observed that subacute exposure to PM_2.5_ could increase HOMA-IR score even on five days of low concentration [11]. A study of 1023 Mexican Americans (17.9–65.6 years) also showed that short-term (up to 58 days) exposure to PM_2.5_ and higher annual average PM_2.5_ exposure were associated with higher serum glucose and HOMA-IR [61]. Another study of 65 nonsmoking adults with metabolic syndrome and IR showed that PM_2.5_ was associated with worsening IR from 2012 to 2013 in Beijing, China [30]. Liu et al. described the biological mechanisms of air pollution (especially PM_2.5_) and IR or diabetes in a systematic review [44], including activation of the central nervous system, alterations in glucose and lipid metabolism, endoplasmic reticulum stress, pulmonary and systemic inflammation, and so on. These results provided an in-depth study of the mechanism of air pollution-mediated IR. However, we did not find any evidence of associations between PM_2.5_ and IR biomarkers in the current study. This discrepancy may be explained through the following points: First, participants in the above three studies had specific population characteristics, i.e., the age and geographical distribution of these studies was limited to specific groups of people and areas; however, in our study, the participants were relatively widely distributed. Secondly, the differences in methodology might also explain the results. The included studies in our research used regression models (*n* = 3), spatial-temporal model (*n* = 2), and U.S. Environmental Protection Agency’s Air Quality System and Federal Reference Method (FRM; *n* = 1) to measure the concentration of air pollutants, which differed from the measurement methods (community monitor [11], tapered element oscillating microbalance [30], multipollutant model [61]) used in those three studies. Moreover, it was difficult to exclude the influence of population susceptibility (such as obesity and immune resistance) on the outcome [11]. People from different races and regions have different gene susceptibility and lifestyle that lead to different degree of physical change with the same level of air pollution, and this difference may even be seen in the group with the same race and region. Furthermore, the contribution of environmental determinants other than air pollutants on IR cannot be ignored [11,30]. Alveolar macrophages and bronchial epithelial cells are the initial cellular sensors of matter, and they do not respond to PM itself but to the biological components of PM, such as lipopolysaccharide (LPS). LPS concentration is higher in PM_10_ than in PM_2.5_, which might explain our findings that IR was not sensitive to the concentration changes of PM_2.5_.

This meta-analysis found no significant relationships between air pollutants and glucose, HbA1c, and leptin. A number of studies have shown that long-term exposure to traffic pollution or particulate air pollution may be associated with increased level of glucose and HbA1c [14,39,62,63]. However, participants with diabetes or prediabetes might contribute to this positive effect [14]. Although some short-term exposure to air pollutants may be associated with evidence of higher levels of glucose, this association may not be detected in long-term exposure. In addition, the age distribution of the target population included in this paper was wide, and the adolescents and young adults among them were less sensitive to the exposure to air pollutants, which might have buffered the effect of air pollution. Wang et al. [64] found that distance to roadway was not associated with leptin levels, while the study by Wolf et al. [14] showed annual average NO_x_ and NO_2_ had a positive association with leptin. However, lack of information about what and when the participants ate, the time of study visit, and blood draw might affect the outcome [64], which could also explain the findings in this meta-analysis.

## 5. Conclusions

We found that NO_2_ and PM_10_ can increase the risk of insulin resistance and that there is no association between PM_2.5_ and insulin resistance. Our results conclude that increased exposure to air pollution can lead to insulin resistance, potentially leading to diabetes and cardiometabolic disease. Further study is warranted to confirm these results and to assess the clinical significance of the results.

## Figures and Tables

**Table 1:** Characteristics of included studies.

Author	Thiering et al. [13]	Wolf et al. [14]	Thiering et al. [31]	Madhloum et al. [41]	Alderete et al. [40]	Li et al. [53]
Published Year	2013	2016	2016	2017	2017	2018
Age	10.2±0.2	Average age 56.2	15	Newborns	8~15	Average age 51
Study country	German	Southern Germany	German	Belgium	USA	US
Sample size	397	2944	837	590	314	5958
Study period	10 years	2006–2008	15 years	2010–2014	2001–2012	1998–2011
Exposure	NO_2_, PM_10_, PM_2.5_, per 500 m decrease in distance to major road (m)	PM_2.5_, PM_10_, PM coarse, nitrogen monoxides (NO_x_), NO_2_	NO_2_, PM_10_, PM_2.5_	PM_2.5_, PM_10_, NO_2_	NO_2_, PM_2.5_	PM_2.5_, traffic-related pollution
Exposure assessment (methods)	LUR models were used to estimate long-term spatial variability of NO_2_, PM_10_, PM_2.5_, and PM_2.5_ absorbance at the birth address of each individual. The concentrations of NO_2_ were measured at 40 monitoring sites, and concentrations of PM_2.5_ and filter absorbance of PM_2.5_ were measured at 20 monitoring sites in Munich-Augsburg and the Ruhr area. The measurement period in Munich was between October 2008 and November 2009, and measurements at all selected sites were carried out three times for 14 consecutive days in different seasons.	Air pollution measurements of PM_10_, PM_2.5_, NO_2_, and the sum of NO_2_ and nitrogen monoxides (NO_x_) were collected at 20 (PM) and 40 (NO_x_) monitoring sites for three periods of two weeks in the cold, warm, and one intermediate season during the period from October 2008 to July 2009. LUR models were then applied to the residence addresses of study participants to assess individual long-term concentrations.	Measurements of particulate matter were conducted at 20 monitoring sites distributed throughout each study area for three, two-week periods in cold, warm, and intermediate temperature seasons between October 2008 and July 2009. For NO_2_, parallel measurements using these 20 and additional 20 monitoring sites were performed. The annual mean concentrations of the pollutants were estimated for all residences at the time of the 15-year examination (2011–2014) using the European Study of Cohorts for Air Pollution Effects (ESCAPE) area-specific LUR models.	The regional background levels of air pollutants (PM_2.5_, PM_10_, NO_2_) for each mother’s residential address were interpolated using a spatial temporal interpolation method (Kriging) that uses pollution data collected in the official fixed site monitoring network (*n* = 34) and land-cover data obtained from satellite images (CORINE land-cover data set) in combination with a dispersion model. To explore potentially critical exposures windows during pregnancy, individual mean air pollutant concentrations (micrograms per cubic meter) were calculated for each of the three trimesters of pregnancy, with trimesters being defined as 1–13 weeks (1st trimester), 14–26 weeks (2nd trimester), and 27 weeks to delivery (3rd trimester).	Hourly air quality data from ambient monitoring stations were downloaded from the U.S. Environmental Protection Agency’s Air Quality System (AQS) for the relevant time period and averaged to daily level. Monthly averages were calculated from the daily data using a 75% completeness criterion, and monthly exposure values were spatially interpolated from the air quality monitoring station’s locations to the finest geographic resolution possible (usually parcel-level) based on the participant’s geocoded street level residence using an inverse distance-squared weighting (IDW2) algorithm.	Annual average concentration of PM_2.5_: ArcGIS software and a hybrid spatial-temporal model were used to estimate PM_2.5_ concentration at residential address. Short-term exposure assessment: central-site hourly measure of PM_2.5_ from the Harvard Supersite air pollution monitoring station located on the rooftop of the Francis A. A tapered element oscillating microbalance was used to measure PM_2.5_. Ambient levels of NO_x_ and O_3_ were computed by averaging data collected from local state monitors (three for NO_x_ and two for O_3_) within the Greater Boston area.
Measurement period	October 2008~November 2009	October 2008~July 2009	October 2008~July 2009	Three trimesters of pregnancy: 1–13 weeks (1st trimester), 14–26 weeks (2nd trimester), and 27 weeks to delivery (3rd trimester).	-	-
Outcome (IR)	HOMA-IR, glucose, and fasting insulin	HOMA-IR, serum glucose, insulin, HbA1c, and leptin	HOMA-IR, glucose, and fasting insulin	Plasma insulin	Glucose and insulin	HOMA-IR, fasting glucose, HbA1c, insulin, and leptin
Outcome measurement	Glucose measurements in blood were performed by standard laboratory methods by the individual hospitals. Fasting insulin in serum was measured centrally by a fully mechanized system, LIAISON (DiaSorin, Saluggia, Italy).	Serum glucose was measured using a hexokinase method (GLU Flex; Dade Behring Marburg, Marburg, Germany). Insulin was determined using ELISA kits from Invitrogen (Camarillo, CA). HbA1c was measured with a reverse-phase, cation exchange, high-performance liquid chromatography method (analyzer HA 8160; Menarini Group). Leptin concentrations were assessed using ELISA kits from Mercodia (Uppsala, Sweden).	Glucose measurements in blood were performed by standard laboratory methods by the two individual hospitals. Fasting insulin in serum was measured centrally by a fully mechanized system, LIAISON (DiaSorin, Saluggia, Italy).	Plasma insulin levels (pmol/L) of umbilical cord blood were measured by an electrochemiluminescence immunoassay on a Modular-E170 (Roche, Basel, Switzerland) immunoanalyzer.	Glucose was assayed using a Yellow Springs Instruments analyzer (YSI INC., Yellow Springs, OH). Insulin was assayed using an automated enzyme immunoassay (Tosoh AIA 600 II analyzer, Tosoh Bioscience, Inc., South San Francisco, CA).	Fasting glucose was measured by the hexokinase method twice in each cohort. Insulin was evaluated by commercially available enzyme-linked immunosorbent assay kits from Linco Research (St. Charles, MO) in Third Generation cohort examination 1, and Roche reagents (R&D Systems, Minneapolis, MN) in Offspring cohort examination 8 and Third Generation cohort examination 2. HbA1c was measured by turbidimetric immunoassay in Offspring cohort examination8 and Third Generation cohort examination 2. Leptin was measured using enzyme-linked immunosorbent assay (R&D Systems, Minneapolis, MN) in Third Generation cohort examination 1.
Adjusted factors	1–3, 7 (paternal), 24–29	1–4, 16–18	1–4, 7 (paternal), 9, 11, 19–23	1–4, 7 (paternal), 9, 11, 19–23	2, 10, 15, 33–37	1 (centered), (1 (centered))^2^, 2, 4–8, 9(median), 11–14, 38, 39
NOS quality score	7	7	6	7	6	8

Adjustment factors: 1: age; 2: sex; 3: ethnicity 4: BMI; 5: smoking; 6: alcohol intake; 7: education; 8: occupation; 9: income; 10: social position; 11: physical activity; 12: date of visit; 13: population density; 14: median value of owner occupied housing units; 15: season of testing (warm/cold); 16: waist-to-hip ratio; 17: month of blood withdrawal; 18: selected socioeconomic and lifestyle variables; 19: study area; 20: cohort; 21: secondhand smoke at home; 22: pubertal scale; 23: NDVI; 24: birth weight; 25: study centre; 26:study; 27: study design; 28: puberty status; 29: ETS; 30: parity; 31: gestational age; 32: season at delivery; 33: season at delivery; 34: prior year exposure at each follow-up visit; 35: body fat percentage; 36: study wave; 37: study entry year; 38: pack years; 39: sine and cosine season. LUR: land use regression; BMI: body mass index; NDVI: normalized difference vegetation Index; ETS: environmental tobacco smoke; HbA1c: hemoglobin A1C.

**Table 2:** Egger’s test to explore publication bias for each pollutant.

Pollutants and IR Biomarkers	Percentage change	SE	Z-Egger	*p*-Egger
PM_2.5_ and HOMA-IR	−0.26	0.41	2.91	0.004
PM_2.5_ and glucose	0.02	0.03	1.19	0.23
PM_2.5_ and insulin	2.39	1.57	2.12	0.03
PM_2.5_ and HbA1c	0.00	0.00	1.19	0.24
PM_2.5_ and leptin	0.01	0.01	1.67	0.09
NO_2_ and HOMA-IR	1.25	0.30	2.31	0.02
NO_2_ and glucose	0.04	0.03	1.60	0.11
NO_2_ and insulin	0.60	0.22	2.39	0.02
PM_10_ and HOMA-IR	2.77	1.07	−1.18	0.24
PM_10_ and insulin	2.75	1.17	0.26	0.80

SE (Standard error): The standard error of a statistic (usually an estimate of a parameter) is the standard deviation of its sampling distribution or an estimate of that standard deviation.

## Supplementary Materials

The following are available online at http://www.mdpi.com/1660-4601/15/11/2593/s1, Table S1: Meta-regression of age and IR biomarkers exposure to air pollutants. Table S2: Specifics of quality score of the included studies.

## References

[1] World Health Organization (2005). Air Quality Guidelines: Global Update.

[2] World Health Organization (2014). Methods for Burden of Disease Attributable to Ambient Air Pollution for the Year 2012.

[3] Esposito K., Petrizzo M., Maiorino M.I., Bellastella G., Giugliano D. (2016). Particulate matter pollutants and risk of type 2 diabetes: A time for concern?. Endocrine.

[4] Xu L., Suresh S., Guo H., Weber R.J., Ng N.L. (2015). Aerosol characterization over the southeastern United States using high resolution aerosol mass spectrometry: Spatial and seasonal variation of aerosol composition, sources, and organic nitrates. Atmos. Chem. Phys..

[5] Zhang Q., Jimenez J.L., Canagaratna M.R., Allan J.D., Coe H., Ulbrich I., Alfarra M.R., Takami A., Middlebrook A.M., Sun Y.L. (2007). Ubiquity and dominance of oxygenated species in organic aerosols in anthropogenically-influenced Northern Hemisphere midlatitudes. Geophys. Res. Lett..

[6] Donkelaar A.V., Martin R.V., Brauer M., Boys B.L. (2015). Use of Satellite Observations for Long-Term Exposure Assessment of Global Concentrations of Fine Particulate Matter. Environ. Health Perspect..

[7] Lim S.S., Vos T., Flaxman A.D., Danaei G., Shibuya K., Adair-Rohani H., Amann M., Anderson H.R., Andrews K.G., Aryee M. (2012). A comparative risk assessment of burden of disease and injury attributable to 67 risk factors and risk factor clusters in 21 regions, 1990–2010: A systematic analysis for the global burden of disease study 2010. Lancet.

[8] Cai Y., Zhang B., Ke W., Feng B., Lin H., Xiao J., Zeng W., Li X., Tao J., Yang Z. (2016). Associations of Short-Term and Long-Term Exposure to Ambient Air Pollutants With Hypertension: A Systematic Review and Meta-Analysis. Hypertension.

[9] Renzi M., Cerza F., Gariazzo C., Agabiti N., Cascini S., Di Domenicantonio R., Davoli M., Forastiere F., Cesaroni G. (2017). Air pollution and occurrence of type 2 diabetes in a large cohort study. Environ. Int..

[10] Balti E.V., Echouffotcheugui J.B., Yako Y.Y., Kengne A.P. (2014). Air pollution and risk of type 2 diabetes mellitus: A systematic review and meta-analysis. Diabetes Res. Clin. Pract..

[11] Brook R.D., Xu X., Bard R.L., Dvonch J.T., Morishita M., Kaciroti N., Sun Q., Harkema J., Rajagopalan S. (2013). Reduced metabolic insulin sensitivity following sub-acute exposures to low levels of ambient fine particulate matter air pollution. Sci. Total Environ..

[12] Jin H.K., Hong Y.C. (2012). GSTM1, GSTT1, and GSTP1 Polymorphisms and Associations between Air Pollutants and Markers of Insulin Resistance in Elderly Koreans. Environ. Health Perspect..

[13] Thiering E., Cyrys J., Kratzsch J., Meisinger C., Hoffman B., Berdel D., von Berg A., Koletzko S., Bauer C.P., Heinrich J. (2013). Long-term exposure to traffic-related air pollution and insulin resistance in children: Results from the GINIplus and LISAplus birth cohorts. Diabetologia.

[14] Wolf K., Popp A., Schneider A., Breitner S., Hampel R., Rathmann W., Herder C., Roden M., Koenig W., Meisinger C. (2016). Association Between Long-Term Exposure to Air Pollution and Biomarkers Related to Insulin Resistance, Subclinical Inflammation and Adipokines. Diabetes.

[15] Thomsen C., Storm H., Christiansen C., Rasmussen O.W., Larsen M.K., Hermansen K. (1997). The day-to-day variation in insulin sensitivity in non-insulin-dependent diabetes mellitus patients assessed by the hyperinsulinemic-euglycemic clamp method. Metabolism.

[16] Moberg E., Kollind M., Lins P.E., Adamson U. (1995). Day-to-day variation of insulin sensitivity in patients with type 1 diabetes: Role of gender and menstrual cycle. Diabet. Med..

[17] Matthews D.R., Hosker J.P., Rudenski A.S., Naylor B.A., Treacher D.F., Turner R.C. (1985). Homeostasis model assessment: Insulin resistance and fJ-cell function from fasting plasma glucose and insulin concentrations in man. Diabetologia.

[18] Karne R.J., Chen H., Quon M.J. (2004). Diagnosing insulin resistance by simple quantitative methods in subjects with normal glucose metabolism. Diabetes Care.

[19] Cameron A.J., Magliano D.J., Zimmet P.Z., Welborn T.A., Colagiuri S., Tonkin A.M., Shaw J.E. (2010). The metabolic syndrome as a tool for predicting future diabetes: The AusDiab study. J. Intern. Med..

[20] Morris D.H., Khunti K., Achana F., Srinivasan B., Gray L.J., Davies M.J., Webb D. (2013). Progression rates from HbA 1c 6.0–6.4% and other prediabetes definitions to type 2 diabetes: A meta-analysis. Diabetologia.

[21] Herder C., Roden M. (2011). Genetics of type 2 diabetes: Pathophysiologic and clinical relevance. Eur. J. Clin. Investig..

[22] Olefsky J.M., Glass C.K. (2010). Macrophages, Inflammation, and Insulin Resistance. Annu. Rev. Physiol..

[23] Peters A. (2012). Epidemiology: Air pollution and mortality from diabetes mellitus. Nature Rev. Endocrinol..

[24] Rajagopalan S., Brook R.D. (2012). Air pollution and type 2 diabetes: Mechanistic insights. Diabetes.

[25] Sun Q., Yue P., Deiuliis J.A., Lumeng C.N., Kampfrath T., Mikolaj M.B., Cai Y., Ostrowski M.C., Lu B., Parthasarathy S. (2009). Ambient air pollution exaggerates adipose inflammation and insulin resistance in a mouse model of diet-induced obesity. Circulation.

[26] Anusree S.S., Sindhu G., Preetha M.R., Raghu K.G. (2018). Insulin resistance in 3T3-L1 adipocytes by TNF-α is improved by punicic acid through upregulation of insulin signalling pathway and endocrine function, and downregulation of proinflammatory cytokines. Biochimie.

[27] Völz S., Svedlund S., Andersson B., Gan L.M., Rundqvist B. (2017). Coronary flow reserve in patients with resistant hypertension. Clin. Res. Cardiol..

[28] Gunji T., Matsuhashi N., Sato H., Iijima K., Fujibayashi K., Okumura M., Sasabe N., Urabe A. (2011). Alcohol consumption is inversely correlated with insulin resistance, independent of metabolic syndrome factors and fatty liver diseases. J. Clin. Gastroenterol..

[29] Probsthensch N.M. (2010). Chronic age-related diseases share risk factors: Do they share pathophysiological mechanisms and why does that matter?. Swiss Med. Wkly..

[30] Brook R.D., Sun Z., Brook J.R., Zhao X., Ruan Y., Yan J., Mukherjee B., Rao X., Duan F., Sun L. (2016). Extreme Air Pollution Conditions Adversely Affect Blood Pressure and Insulin Resistance: The Air Pollution and Cardiometabolic Disease Study. Hypertension.

[31] Thiering E., Markevych I., Brüske I., Fuertes E., Kratzsch J., Sugiri D., Hoffmann B., Von Berg A., Bauer C.P., Koletzko S. (2016). Associations of Residential Long-Term Air Pollution Exposures and Satellite-Derived Greenness with Insulin Resistance in German Adolescents. Environ. Health Perspect..

[32] Teichert T., Vossoughi M., Vierkötter A., Sugiri D., Schikowski T., Schulte T., Roden M., Luckhaus C., Herder C., Krämer U. (2013). Association between Traffic-Related Air Pollution, Subclinical Inflammation and Impaired Glucose Metabolism: Results from the SALIA Study. PLoS ONE.

[33] Toledocorral C.M., Alderete T.L., Habre R., Berhane K., Lurmann F.W., Weigensberg M.J., Goran M.I., Gilliland F.D. (2016). Effects of air pollution exposure on glucose metabolism in LosAngeles minority children. Pediatr. Obes..

[34] Wardcaviness C.K., Kraus W.E., Blach C., Haynes C.S., Dowdy E., Miranda M.L., Devlin R.B., Diazsanchez D., Cascio W.E., Mukerjee S. (2015). Association of Roadway Proximity with Fasting Plasma Glucose and Metabolic Risk Factors for Cardiovascular Disease in a Cross-Sectional Study of Cardiac Catheterization Patients. Environ. Health Perspect..

[35] Li H., Cai J., Chen R., Zhao Z., Ying Z., Wang L., Chen J., Hao K., Kinney P.L., Chen H. (2017). Particulate Matter Exposure and Stress Hormone Levels: A Randomized, Double-Blind, Crossover Trial of Air Purification. Circulation.

[36] Møller P., Loft S. (2010). Oxidative Damage to DNA and Lipids as Biomarkers of Exposure to Air Pollution. Environ. Health Perspect..

[37] Lodovici M., Bigagli E. (2011). Oxidative Stress and Air Pollution Exposure. J. Toxicol..

[38] U.S. Environmental Protection Agency (2016). Integrated Science Assessment for Oxides of Nitrogen—Health Criteria.

[39] Kelishadi R., Mirghaffari N., Poursafa P., Gidding S.S. (2009). Lifestyle and environmental factors associated with inflammation, oxidative stress and insulin resistance in children. Atherosclerosis.

[40] Alderete T.L., Habre R., Toledocorral C.M., Berhane K., Chen Z., Lurmann F.W., Weigensberg M.J., Goran M.I., Gilliland F.D. (2017). Longitudinal Associations Between Ambient Air Pollution with Insulin Sensitivity, β-Cell Function, and Adiposity in Los Angeles Latino Children. Diabetes.

[41] Madhloum N., Janssen B.G., Martens D.S., Saenen N.D., Bijnens E., Gyselaers W., Penders J., Vanpoucke C., Lefebvre W., Plusquin M. (2017). Cord plasma insulin and in utero exposure to ambient air pollution. Environ. Int..

[42] Sørensen M., Daneshvar B., Hansen M., Dragsted L.O., Hertel O., Knudsen L., Loft S. (2003). Personal PM2.5 exposure and markers of oxidative stress in blood. Environ. Health Perspect..

[43] Jerrett M., Brook R., White L.F., Burnett R.T., Yu J., Su J., Seto E., Marshall J., Palmer J.R., Rosenberg L. (2017). Ambient ozone and incident diabetes: A prospective analysis in a large cohort of African American women. Environ. Int..

[44] Liu C., Ying Z., Harkema J., Sun Q., Rajagopalan S. (2013). Epidemiological and experimental links between air pollution and type 2 diabetes. Toxicol. Pathol..

[45] Xu X., Liu C., Xu Z., Tzan K., Zhong M., Wang A., Lippmann M., Chen L.C., Rajagopalan S., Sun Q. (2011). Long-term exposure to ambient fine particulate pollution induces insulin resistance and mitochondrial alteration in adipose tissue. Toxicol. Sci..

[46] Zheng Z., Xu X., Zhang X., Wang A., Zhang C., Hüttemann M., Grossman L.I., Chen L.C., Rajagopalan S., Sun Q. (2013). Exposure to Ambient Particulate Matter Induces a NASH-like Phenotype and Impairs Hepatic Glucose Metabolism in an Animal Model. J. Hepatol..

[47] Hutton B., Salanti G., Caldwell D.M., Chaimani A., Schmid C.H., Cameron C., Ioannidis J.P.A., Straus S., Thorlund K., Jansen J.P. (2015). The PRISMA Extension Statement for Reporting of Systematic Reviews Incorporating Network Meta-analyses of Health Care Interventions: Checklist and Explanations. Ann. Intern. Med..

[48] Wells G.A., Shea B.J., O’Connell D., Peterson J., Welch V., Losos M., Tugwell P. (2012). The Newcastle–Ottawa Scale (NOS) for Assessing the Quality of Non-Randomized Studies in Meta-Analysis. A Appl. Eng. Agric..

[49] Dersimonian R., Laird N. (1986). Meta-analysis in clinical trials. Control. Clin. Trials.

[50] Normand S.L. (1999). Meta-analysis: Formulating, evaluating, combining, and reporting. Stat. Med..

[51] Thompson S.G., Sharp S.J. (1999). Explaining heterogeneity in meta-analysis: A comparison of methods. Control. Clin. Trials.

[52] Stuck A.E., Rubenstein L.Z., Wieland D., Vandenbroucke J.P., Irwig L., Macaskill P., Berry G., Glasziou P., Seagroatt V., Stratton I. (1997). Bias in Meta-Analysis Detected by a Simple, Graphical Test. BMJ.

[53] Li W., Dorans K.S., Wilker E.H., Rice M.B., Kloog I., Schwartz J.D., Koutrakis P., Coull B.A., Gold D.R., Meigs J.B. (2017). Ambient air pollution, adipokines, and glucose homeostasis: The Framingham Heart Study. Environ. Int..

[54] Sabin M.A., Magnussen C.G., Juonala M., Shield J.P., Kähönen M., Lehtimäki T., Rönnemaa T., Koskinen J., Loo B.M., Knip M. (2015). Insulin and BMI as predictors of adult type 2 diabetes mellitus. Pediatrics.

[55] Li C., Fang D., Xu D., Wang B., Zhao S., Yan S., Wang Y. (2014). Main air pollutants and diabetes-associated mortality: A systematic review and meta-analysis. Eur. J. Endocrinol..

[56] Doi S.A., Barendregt J.J., Williams G.M., Khan S., Thalib L. (2015). Simulation Comparison of the Quality Effects and Random Effects Methods of Meta-analysis. Epidemiology.

[57] Fleisch A.F., Luttmann-Gibson H., Perng W., Rifas-Shiman S.L., Coull B.A., Kloog I., Koutrakis P., Schwartz J.D., Zanobetti A., Mantzoros C.S. (2016). Prenatal and early life exposure to traffic pollution and cardiometabolic health in childhood. Pediatr. Obes..

[58] Park S.K., Wang W. (2014). Ambient Air Pollution and Type 2 Diabetes Mellitus: A Systematic Review of Epidemiologic Research. Curr. Environ. Health Rep..

[59] Eze I.C., Imboden M., Kumar A., Von E.A., Stolz D., Gerbase M.W., Künzli N., Pons M., Kronenberg F., Schindler C. (2016). Air pollution and diabetes association: Modification by type 2 diabetes genetic risk score. Environ. Int..

[60] Coogan P.F., White L.F., Yu J., Burnett R.T., Marshall J.D., Seto E., Brook R.D., Palmer J.R., Rosenberg L., Jerrett M. (2016). Long term exposure to NO_2_ and diabetes incidence in the Black Women’s Health Study. Environ. Res..

[61] Chen Z., Salam M.T., Toledocorral C., Watanabe R.M., Xiang A.H., Buchanan T.A., Habre R., Bastain T.M., Lurmann F., Wilson J.P. (2016). Ambient Air Pollutants Have Adverse Effects on Insulin and Glucose Homeostasis in Mexican Americans. Diabetes Care.

[62] Chuang K.J., Yan Y.H., Cheng T.J. (2010). Effect of air pollution on blood pressure, blood lipids, and blood sugar: A population-based approach. J. Occup. Environ. Med..

[63] Chuang K.J., Yan Y.H., Chiu S.Y., Cheng T.J. (2011). Long-term air pollution exposure and risk factors for cardiovascular diseases among the elderly in Taiwan. Occup. Environ. Med..

[64] Wang Y., Eliot M.N., Kuchel G.A., Schwartz J., Coull B.A., Mittleman M.A., Lipsitz L.A., Wellenius G.A. (2014). Long-term exposure to ambient air pollution and serum leptin in older adults: Results from the MOBILIZE Boston study. J. Occup. Environ. Med..

