# Supplementary material for: Associations of Exposure to Air Pollution with Insulin Resistance: A Systematic Review and Meta-Analysis
Source: Int J Environ Res Public Health. 2018 Nov 20;15(11):2593. doi: 10.3390/ijerph15112593 (PMC6266153; doi:10.3390/ijerph15112593)
Supplement: Supplementary file 1 [file ijerph-15-02593-s001.pdf]

Table S1. Meta-regression of age and IR biomarkers exposure to air pollutants.

Pollutants and IR biomarkers	% change	95%CI	SE	z-value	p-value
$PM_{2.5}$ and HOMA-IR	0.0378	(-0.6293, 0.7048)	0.3403	0.1110	0.9116
$PM_{2.5}$ and glucose	0.0088	(-0.0259, 0.0435)	0.0177	0.4957	0.6201
$PM_{2.5}$ and insulin	0.3130	(-0.5472, 1.1733)	0.4389	0.7132	0.4757
$PM_{2.5}$ and HbA1c	0.1069	(-0.1006, 0.3145	0.1059	1.0097	0.3126
$PM_{2.5}$ and leptin	1.8871	(-1.6166, 5.3908	1.7876	1.0556	0.2911
NO_2 and HOMA-IR	0.5132	(-0.6013, 1.6277	0.5686	0.9026	0.3668
NO_2 and glucose	0.0356	(-0.0084, 0.0795	0.0224	1.5864	0.1126
NO_2 and insulin	0.6673	(-0.8950, 2.2296	0.7971	0.8371	0.4025
PM_{10} and HOMA-IR	0.1624	(-0.6078, 0.9326	0.3930	0.4133	0.6794

SE (Standard error): The standard error of a statistic (usually an estimate of a parameter) is the standard deviation of its sampling distribution or an estimate of that standard deviation.

Table S2. Specifics of quality score of the included studies.

	Thiering et al. (2013)	Wolf et al. (2016)	Thiering et al. (2016)	Madhlouma et al. (2017)	Alderete et al. (2017)	Li et al. (2018)
NOS quality score	7	7	6	7	6	8
**Selection**						
**1) Representativeness of the exposed cohort**<br>a) truly representative of the average ________ (describe) in the community<br>b) somewhat representative of the average ________ in the community<br>c) selected group of users eg nurses, volunteers<br>d) no description of the derivation of the cohort	b	b	c	c	c	b
**2) Selection of the non-exposed cohort**<br>a) drawn from the same community as the exposed cohort	b	a	b	a	a	a

b) drawn from a different source<br>c) no description of the derivation of the non-exposed cohort						
**3) Ascertainment of exposure**<br>a) secure record (eg surgical records)<br>b) structured interview<br>c) written self-report<br>d) no description	a	a	a	a	a	a
**4) Demonstration that outcome of interest was not present at start of study**<br>a) yes<br>b) no	a	b	a	a	a	a
**Comparability**						
**1) Comparability of cohorts on the basis of the design or analysis**<br>a) study controls for __________ (select the most important factor)<br>b) study controls for any additional factor (This criteria could be modified to indicate specific control for a second important factor.)	b	b	b	b	b	b
**Outcome**						
**1) Assessment of outcome**<br>a) independent blind assessment<br>b) record linkage<br>c) self-report<br>d) no description	a	a	a	a	a	a
**2) Was follow-up long enough for outcomes to occur**<br>a) yes (select an adequate follow up period for outcome of interest)<br>b) no	a	a	a	a	a	a

**3) Adequacy of follow up of cohorts**<br>a) complete follow up - all subjects accounted for<br>**b)** subjects lost to follow up unlikely to introduce bias - small number lost - > ____ % (select an adequate %) follow up, or description provided of those lost)<br>c) follow up rate < ____% (select an adequate %) and no description of those lost<br>d) no statement	b	b	b	b	d	b
